# Genome-Wide Identification of *BPC* Gene Family in Ten Cotton Species and Function Analysis of *GhBPC4* Involved in Cold Stress Response

**DOI:** 10.3390/ijms26167978

**Published:** 2025-08-18

**Authors:** Faren Zhu, Qing Xu, Jiliang Fan, Lu Meng, Rong Wang, Jiahuan Niu, Jingru Wang, Ganggang Zhang, Shandang Shi, Fei Wang, Hongbin Li

**Affiliations:** Key Laboratory of Oasis Town and Mountain-Basin System Ecology of Xinjiang Production and Construction Corps, Key Laboratory of Xinjiang Phytomedicine Resource and Utilization of Ministry of Education, College of Life Sciences, Shihezi University, Shihezi 832003, China; zhufaren163@163.com (F.Z.); xq19901526963@163.com (Q.X.); fanplanbeson@163.com (J.F.); mlulu@stu.shzu.edu.cn (L.M.); wangrongbio@163.com (R.W.); 17599931126@163.com (J.N.); 18935768685@163.com (J.W.); 19915233386@163.com (G.Z.); shi_shandang@163.com (S.S.)

**Keywords:** cotton, BPC transcription factor family, genome-wide identification, cold stress

## Abstract

Basic Pentacysteine (BPC) represents a class of plant-exclusive transcription factors, serving pivotal roles in orchestrating developmental processes and mediating responses to both biotic and abiotic stressors. However, the genome-wide characteristics and low-temperature response mechanism of the *BPC* gene family in cotton remain unclear. Employing a genome-wide screening approach, this study characterized 60 distinct BPC transcription factor genes across ten *Gossypium* species. Conserved structural analysis showed that all *BPC* members carried a highly conserved GAGA-binding domain. Concurrently, the exploration of *cis*-acting elements within promoter regions demonstrated the potential involvement of these BPC transcription factors in modulating developmental processes, hormone signaling cascades, and abiotic stress adaptation mechanisms. Genomic collinearity analysis shows that segmental duplication is the core mechanism for the expansion of this gene family. Expression analysis indicated that the transcription level of *GhBPC4* was significantly increased under low-temperature stress. Genetic function studies displayed that VIGS-mediated *GhBPC4* silencing reduced cotton cold tolerance. This study systematically analyzed the genomic characteristics of the cotton BPC transcription factor family and functionally validated the molecular mechanism of *GhBPC4*-mediated cryogenic response. These findings establish an important foundation for subsequent analysis of multidimensional regulatory networks and the breeding of cold-resistant cotton germplasms.

## 1. Introduction

Cotton (*Gossypium* spp.) serves as a fundamental raw material for the global textile industry, and its natural fibers play a crucial strategic role in the national economy [1]. As a typical heat-sensitive crop, cotton is predominantly cultivated across tropical and subtropical regions worldwide [2]. However, its growth and development processes are highly sensitive to temperature fluctuations. Affected by global climate change and adjustment of cultivated land patterns, cotton planting areas are gradually expanding to high latitudes, making plants more vulnerable to abiotic stresses like low temperature during the growth stage [3]. Cold stress will significantly delay seed germination, inhibit seedling growth, reduce the development quality of cotton bolls, and eventually cause yield loss. Therefore, in-depth analysis of the molecular regulatory mechanisms in cotton’s response to cold stress, and the cultivation of new cold-resistant cotton germplasms on this basis, has become the key goal of current cotton genetic improvement research.

BPC (Basic Penta Cysteine) is a class of plant-specific DNA-binding proteins. Its core feature is that the C-terminals contain a conserved *BPC* domain (about 98 amino acids), which forms intramolecular disulfide bonds through five cysteine residues (C1-C5), stabilizes its three-dimensional conformation, and enhances its binding ability to GA/TC repeats [4,5]. In *Arabidopsis thaliana*, the seven members of this family can be systematically divided into three subclasses according to sequence conservation and N-terminal domain differences: Class I includes *BPC1*, *BPC2,* and *BPC3*, with a typical Penta Cysteine motif; Class II includes *BPC4*, *BPC5*, and *BPC6*, whose N-terminal contains the L-helix predictive structure, in which *BPC5* may be a functionally inactive pseudogene due to an in-frame stop codon; and Class III contains only one member, *BPC7*, which has the highest sequence uniqueness and low homology with the other two classes [6,7]. The BPC transcription factor regulates related genes through epigenetic modification. Early studies have confirmed that it promotes the trimethylation of histone H3K27 (H3K27me3) by recruiting Polycomb inhibitor complex 2 (PRC2) and participates in the regulation of homologous gene expression [8,9]. Subsequent studies have further revealed the complexity of its function as a chromatin boundary element, which cooperates with bHLH transcription factors to maintain topological associative architecture domain (TAD) boundaries and affect three-dimensional genome structure, and interacts with bHLH factors such as SPCH and MUTE to inhibit excessive proliferation of stomatal lineages [10]. The combination of multiple *bpc* mutants exhibits a strong phenotype with extensive defects, playing an important role in plant development [11,12]. In addition, *BPC* genes are actively involved in hormonal and stress responses. *BPC6* directly binds to key BR signaling pathway gene (such as *BZR1*) promoters to regulate BR homeostasis [13]. Maize *BPC* family genes show significant changes in expression after SA, JA, and ethylene treatment, suggesting that they may activate stress response genes through ABA and JA signaling pathway integration; *BPC1* and *BPC6* bind to ethylene-related promoters (such as potato *StBB* gene) [14]. Under salt stress, *BPC1*/*BPC2* promoted galactan accumulation by inhibiting the expression of *AtGALS1* (β-1,4-galactan synthase 1), thereby enhancing cell wall stability and Na^+^ isolation ability [15,16]. In addition, cucumber *CsBPC2* enhances salt tolerance by regulating ion transport and antioxidant genes [17]. In rapeseed, *BPC6* regulates the fatty acid elongase gene, affects epidermal wax deposition, and forms a physical stress barrier [8]. The *BPC* family also enhances plant stress adaptability through a dual regulatory mechanism. *BPC1*/*BPC2*/*BPC4*/*BPC6* directly bind to the *ABI4* promoter, inhibit its expression, and regulate root structural plasticity [18]. In addition, *BPC6* is specifically upregulated in *A*. *thaliana* under cold stress [18]. The expression of *SlBPC2* in tomato increased sharply after 3 h of low-temperature treatment [19], and it was revealed that members of this family have cross-species functional conservation in low-temperature responses.

As key plant regulators, the functions of *BPC* family members in model organisms such as *A. thaliana* have been deeply elucidated [5,9,20,21]. However, the genome-wide systematic characterization of the cotton BPC transcription factor family and its molecular regulatory mechanism under cold stress remain unclear. This study was the first to conduct genome-wide analysis of the BPC transcription factor family across ten cotton species. Based on the whole-genome data, the *BPC* gene family members in each cotton species were successfully identified; then the gene structure characteristics, conserved domain distribution, and *cis*-acting element composition in the promoter region were thoroughly analyzed, and the chromosome location and gene collinearity were clarified. At the same time, the expression profiles of *GhBPC* genes in upland cotton across different tissues and under various stress treatments were analyzed; the biological function of *GhBPC4* in cotton cold stress response was further validated using VIGS technology. This study provides dual support for elucidating the mechanism of cotton cold stress response with evolutionary and functional molecular evidence.

## 2. Results

### 2.1. Identification and Chromosome Distribution of BPC Gene Family

**Figure 1:**
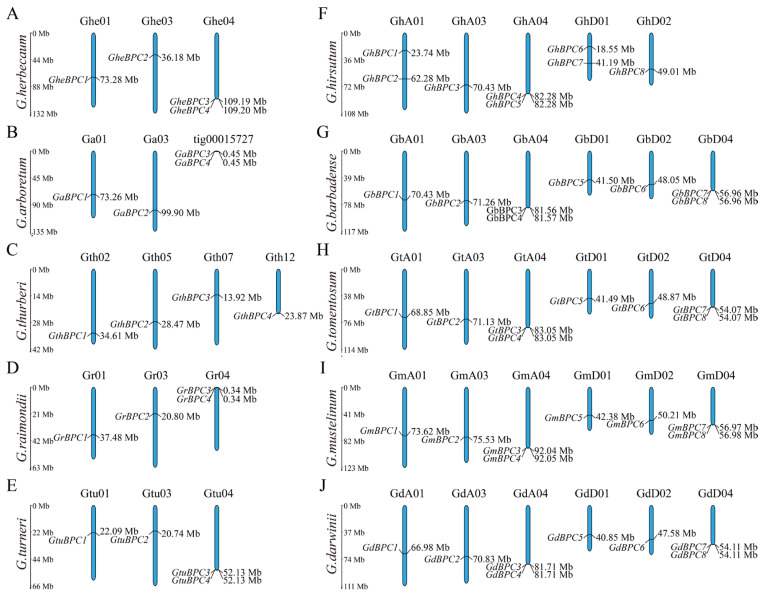
The chromosome localization and distribution characteristics of the *BPC* gene family in 10 *Gossypium* species. (**A**–**J**) The chromosomes of *G. herbaceum* (Ghe), *G. arboretum* (Ga), *G. thurberi* (Gth), *G. raimondii* (Gr), *G. turneri* (Gtu), *G. hirsutum* (Gh), *G. barbadense* (Gb), *G. tomentosum* (Gt), *G. mustelinum* (Gm), and *G. darwinii* (Gd). The vertical scale positioned along the left margin denotes chromosomal lengths in Mb.

To identify *BPC* gene family members across 10 *Gossypium* species, the *AtBPC* protein sequence served as a query for Blastp screening against corresponding cotton genomes, enabling systematic homology mining (E-value was set to 1.0 × 10^−20^). Through genomic sequence screening and redundant sequence elimination, a total of 60 members of the *BPC* gene family were identified in the *Gossypium* genome in this study. Four *BPC* genes were found in *G. herbaceum*, *G. arboretum*, *G. thurberi*, *G. raimondii*, and *G. turneri*, respectively. Tetraploid cotton, *G. hirsutum*, *G. barbadense*, *G. tomentosum*, *G. mustelinum*, and *G. darwinii* each contain eight *BPC* genes. *BPC* family genes were named based on chromosomal positions, and they were designated as *GheBPC1-4*, *GaBPC1-4*, *GthBPC1-4*, *GrBPC1-4*, *GtuBPC1-4*, *GhBPC1-8*, *GbBPC1-8*, *GtBPC1-8*, *GmBPC1-8*, and *GdBPC1-8*. Chromosomal distribution revealed substantial interspecies variations in *BPC* gene copy numbers and spatial distributions among *Gossypium* species, and *BPC* genes showed a concentrated distribution in specific regions of chromosomes (Figure 1). In diploid cotton, the distribution pattern of the four *BPC* genes on the chromosomes of *G. herbaceum*, *G. raimondii*, and *G. turneri* was the same: chromosomes 1 and 3 each carried one gene and chromosome 4 contained two genes. The four genes of *G. thurberi* are evenly distributed on chromosomes 2, 3, 7, and 12. As far as tetraploid cotton is concerned, the distribution of *BPC* genes in *G. hirsutum* is as follows: subgroup A has two genes each on chromosomes 1 and 4, and chromosome 3 has one gene; subgroup D has two genes on chromosome 1 and one gene on chromosome 2. In contrast, the chromosome distribution patterns of *G. barbadense*, *G. tomentosum*, *G. mustelinum*, and *G. darwinii* were completely consistent, as follows: subgroup A had one gene for chromosomes 1 and 3, and two genes for chromosome 4; subgroup D had one gene for chromosomes 1 and 2, and two genes for chromosome 4.

The protein sequences and physicochemical properties of the different BPC transcription factors were significantly different (Table S1): the encoded amino acid sequences ranged from 234 aa (*GthBPC1*) to 336 aa (*GhBPC6*) in length; the molecular weight ranged from 25.46 kDa (*GthBPC1*) to 37.16 kDa (*GhBPC6*); and the isoelectric point ranged from 9.18 (*GrBPC2*) to 9.99 (*GbBPC8*). The instability index of all members exceeded 40, which means that they belong to unstable proteins; the fat coefficient was in the range of 56.72 (*GrBPC3*) to 64.85 (*GhBPC6*); and the hydrophilicity index of all proteins was negative, indicating that they had hydrophilicity characteristics.

### 2.2. Evolutionary Analysis of BPC Gene Family

**Figure 2:**
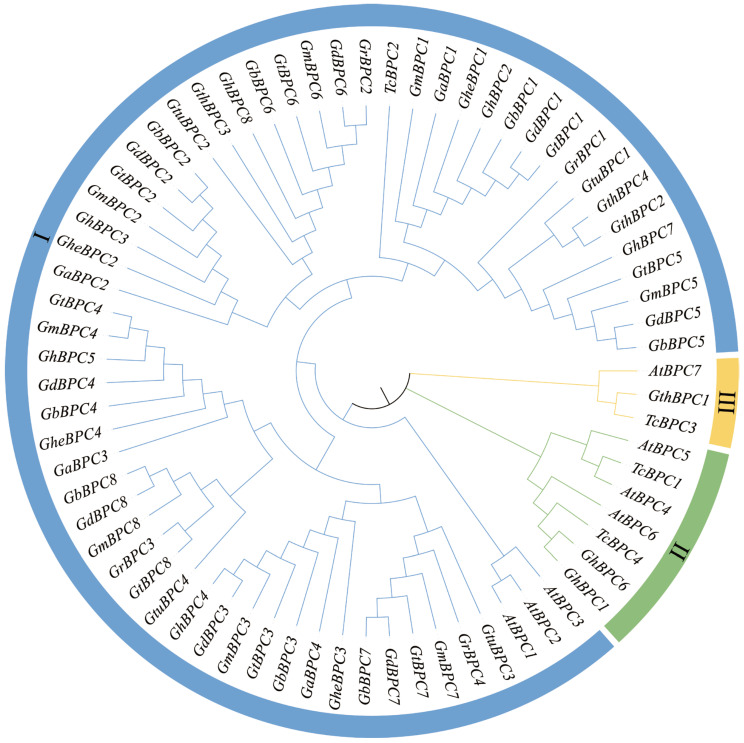
Phylogenetic tree construction for *BPC* gene families in *Arabidopsis thaliana*, ten *Gossypium* species, and *Theobroma cacao* L. The phylogenetic tree was constructed by Neighbor-Joining (NJ). The branches of the NJ tree are color-coded according to subfamily membership.

To elucidate the evolutionary trajectories and phylogenetic relationships of the *BPC* gene family, we selected *A. thaliana* and *Theobroma cacao* L. as key reference species for comparative analysis. *A. thaliana* was selected as a model plant due to its well-annotated genome and extensive stress response mechanisms, which provide a universal framework for studying gene family evolution. Meanwhile, the tropical woody cash crop *Theobroma cacao* L. exhibits profound homology in stress adaptation pathways and ecological relevance to cotton, making it a valuable comparative species for understanding lineage-specific adaptations. Based on the above considerations, we performed multiple sequence alignment of BPC protein amino acid sequences from *A. thaliana*, ten *Gossypium* species, and *Theobroma cacao* L. using Clustal W, followed by Neighbor-Joining (NJ) tree construction (Figure 2). The seventy-one *BPC* genes were classified into three subfamilies. Group I contained 61 members, which was the largest subfamily and highly conserved with *A. thaliana* Class I *BPC* genes. Group II included *GhBPC1* and *GhBPC6*, which had significant homology with *A. thaliana* Class II *BPC* genes. Group III contained only a single member, *GthBPC1*. Phylogenetic reconstruction demonstrates functional divergence among *BPC* genes during evolution. Crucially, the pronounced expansion of group A genes implicates whole-genome duplication and segmental duplication events as primary drivers for the diversification of the cotton *BPC* family.

### 2.3. Analysis of Gene Structure and Conserved Motif and Domains of BPC Gene Family

**Figure 3:**
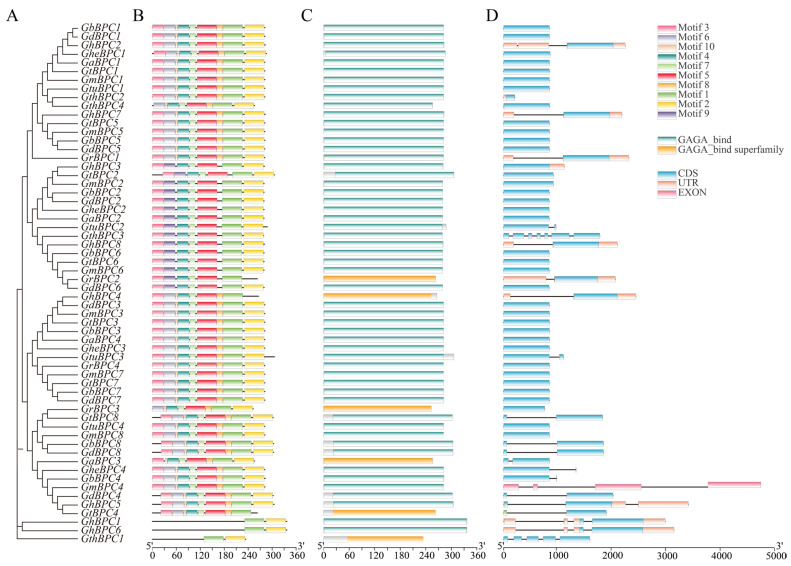
Sequence characteristics of *BPC* genes in ten cotton species. (**A**) Cotton *BPC* genes’ phylogenetic tree. (**B**) Identification of conserved protein motifs (1–10), visualized as distinctively colored boxes. (**C**) Characterization of conserved functional domains in BPC protein sequences. (**D**) Visualization map of *BPC* gene structure: exons (green boxes), introns (black lines), and untranslated regions (yellow boxes).

To comprehensively investigate the molecular attributes of the cotton *BPC* gene family, an integrated methodology leveraging the MEME motif analysis toolkit, Conserved Domain Database, and TBtools bioinformatics software was employed to concurrently assess structural features, conserved domains, and motif patterns across ten *Gossypium* species (Figure 3). The conserved motif distribution shows that motif 1 is ubiquitous in all BPC proteins, indicating that it is the core functional element of this family (Figure 3B). The motif configuration within the subfamily is highly consistent: histone A contains six high-frequency motifs (1–5 and 7), while groups B and C retain only motifs 1 and 2. The differences in motif composition between groups A and B/C confirmed phylogenetic grouping. Analysis of gene structure revealed that UTR distribution was specific, with only 11 genes containing untranslated regions (Figure 3D). In addition, most genes contained 1–2 introns, except *GthBPC1* and *GthBPC3*.

### 2.4. Profiling of Cis-Acting Motifs Within BPC Gene Promoter

**Figure 4:**
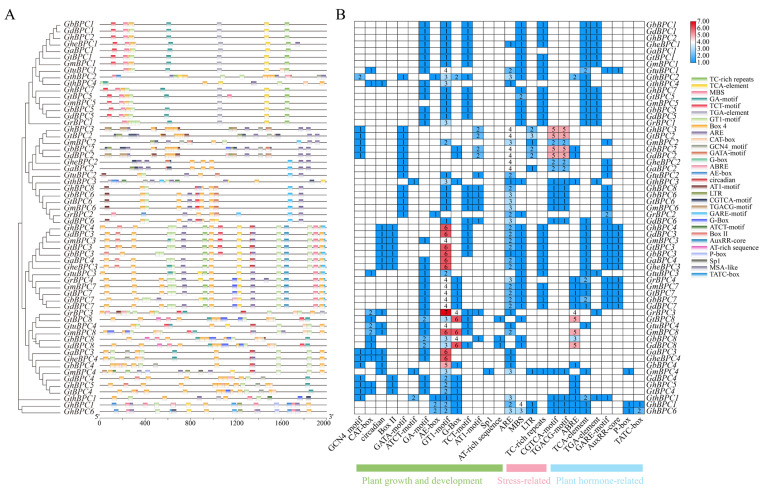
Characterization of *cis*-acting elements of *BPC* gene family. (**A**) Spatial organization of *cis*-regulatory elements within promoter regions, annotated by color-coded boxes denoting distinct functional motifs. (**B**) Profiling of *cis*-element abundance across gene family members via heatmap matrix, where gradient shading and numerical labels indicate element-specific counts.

To decipher the transcriptional regulatory architecture and biological roles of cotton *BPC* genes, computational identification of *cis*-regulatory elements was conducted across the 2000 bp promoter region upstream from the initiation codon (ATG) (Figure 4). Developmental regulatory elements (GA-motif, G-box, GT1-motif, TCT-motif) showed high-frequency enrichment, stress-responsive elements (MBS, ARE) were widely present, and hormone-responsive elements (CGTCA-motif, ABRE, TGACG-motif) were significantly enriched. Although developmental-stress-hormone-related elements were enriched as a whole, there were differences between groups, member specificity, and evolutionary relevance. TCT-motif was enriched in group A and absent in group B; *GmBPC4* contained unique Sp1 elements. The GT1-motif serves as a pivotal regulator in plant transcriptional networks, mainly mediating the responses to biotic invasion and abiotic stress. Differentiated combinations of hormone response elements drive subfamily-specific strategies to reconcile the balance between defense needs and developmental pathways.

### 2.5. Collinearity Analysis of BPC Gene Family

**Figure 5:**
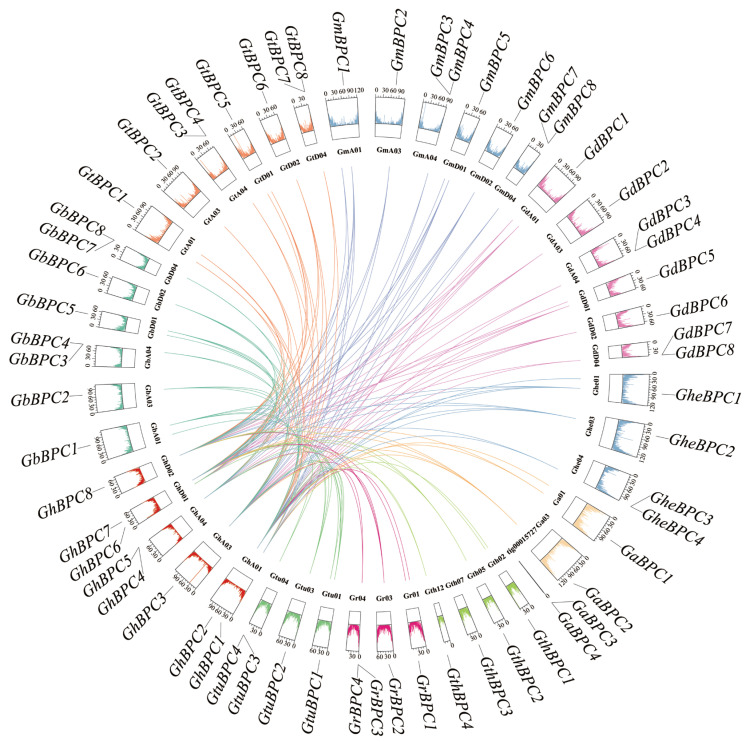
Collinearity between *G. hirsutum* and nine cotton species based on homologous gene pair analysis. Collinear genes linked by different colors. Color blocks represent gene density of various genes and chromosomes.

Based on MCScanX collinearity analysis, this study investigated the expansion mechanism of cotton *BPC* gene family, and systematically revealed the genomic homology between *G. hirsutum* and nine other cotton species. The analysis results showed that the collinear relationship of *GhBPC* genes in *G. hirsutum* was as follows: 11, 7, 7, 8, and 12 pairs of collinearity genes were formed with the *GheBPC*, *GaBPC*, *GthBPC*, *GrBPC*, and *GtuBPC* genes, respectively; 21, 22, 22, and 22 pairs of collinear genes were formed with the other tetraploid cotton species (*GbBPC*, *GtBPC*, *GmBPC*, and *GdBPC*), respectively, indicating that the genome structure of tetraploid cotton is highly conserved among species (Figure 5). The differences in the number of homologous pairs reflect the differentiation effects of the polyploidization history of *Gossypium*.

### 2.6. GhBPC Genes Expression Pattern Analysis

**Figure 6:**
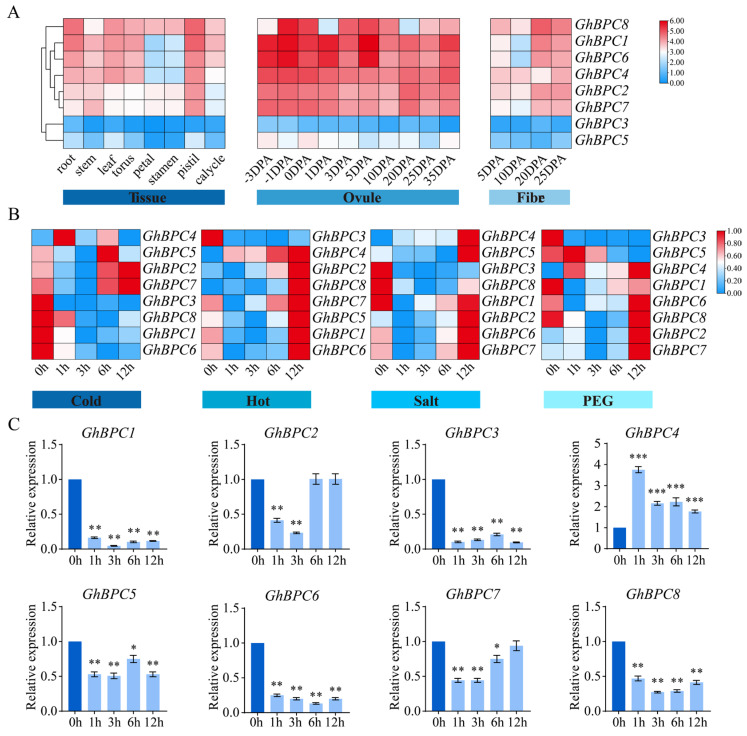
Expression profiling of *GhBPC* genes in *G. hirsutum*. (**A**) Spatiotemporal expression patterns of *GhBPC* family members characterized across tissue development, ovule formation, and fiber differentiation stages. (**B**) Transcriptional dynamics of *GhBPC* genes under abiotic stressors. Heatmap visualization quantifies expression levels via log_2_(FPKM+1) normalization, with blue-to-red color gradient indicating low-to-high relative expression. (**C**) RT-qPCR analysis of *GhBPC* genes under cold stress (4 °C). Error bar represents ± SD (*n* = 3). Significance analysis by one-way ANOVA, with statistical significance denoted by * *p* < 0.05, ** *p* < 0.01, and *** *p* < 0.001.

To elucidate the biological roles of *GhBPC* genes, this research employed transcriptomic profiling to characterize the spatiotemporal regulation patterns of eight *GhBPC* members across distinct tissues and under diverse abiotic stresses. Key functional genes were subsequently identified through systematic screening. Analysis revealed that the transcriptional dynamics of *GhBPC* genes demonstrated variations across multiple tissue types (Figure 6A). *GhBPC1* exhibited markedly elevated expression in the roots, stems, torus, pistils, and calycles relative to other family members. Conversely, *GhBPC8* displayed predominance in leaves, petals, and stamens. Furthermore, transcript abundance of both *GhBPC1* and *GhBPC6* in ovules exceeded that of other genes. *GhBPC3* and *GhBPC5* showed lower expression levels in all tested tissues. The expression profiles of *GhBPC* family members under various abiotic stress conditions (including low temperature, high temperature, salinity, and drought) revealed their dynamic transcriptional responses to environmental stress. (Figure 6B). Under cold stress, the expression levels of *GhBPC2*, *GhBPC4*, *GhBPC5*, and *GhBPC7* were significantly higher than those of other members, among which *GhBPC4* responded the fastest (activated within 1 h); under heat stress, except for *GhBPC3*, the expression levels of the other genes showed an upregulation trend, reaching the expression peak synchronously at 12 h; under salt stress, the expression levels of *GhBPC2*, *GhBPC4*, *GhBPC5*, *GhBPC6*, and *GhBPC7* showed an upregulation trend and formed a co-expression peak at 12 h, but the peak distributions were different; under drought stress, the transcriptional activation of *GhBPC5* was the fastest (within 1 h), while the expression levels of *GhBPC2* and *GhBPC7* showed an upregulation trend and reached a peak at 12 h.

To demonstrate the biological function of *GhBPC* genes in response to low-temperature stress, this study used the RT-qPCR technique to analyze its transcriptional regulatory characteristics under cold stress, and the results showed that the expression level of *GhBPC4* under cold treatment was significantly higher than that of other *BPC* genes. Notably, compared to the control, *GhBPC4* expression showed a 3.8-fold rapid upregulation within 1 h after cold treatment (*p* < 0.001), indicating its potential role as an early responder to low-temperature stress (Figure 6C). Therefore, *GhBPC4* was selected for functional analysis.

### 2.7. Experimental Validation of Subcellular Localization Prediction for GhBPC4

**Figure 7:**
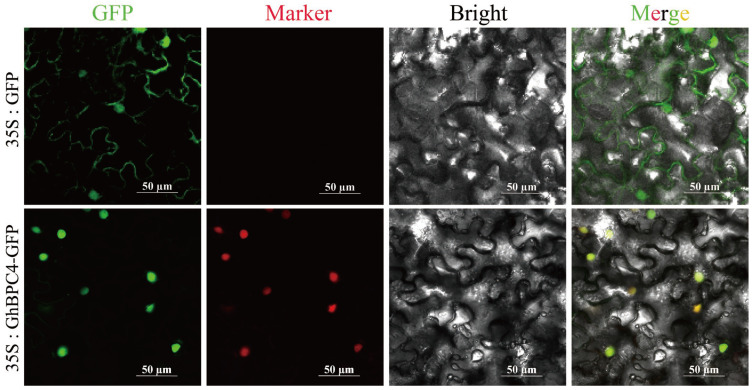
The subcellular distribution of GhBPC4. A 35S promoter-driven recombinant construct (*35S:GhBPC4-GFP*) was generated, with the *35S:GFP* empty vector as the control. Images were displayed under a green fluorescent field, red fluorescent field (nuclear localization), brightfield, and composite field. The scale bar represents 50 µm.

To validate the predicted subcellular localization of GhBPC4 (Cell-PLoc 2.0), a 35S:*GhBPC4*-GFP fusion expression vector was constructed and analyzed by the tobacco leaf transient transformation system. During the experiment, we randomly selected at least 10 different fields of view for each sample for imaging. At the same time, to ensure the reliability of the localization results, we conducted three independent biological replicates, and consistent localization patterns were observed in multiple cells in each replication. Green fluorescent protein (35S:GFP) in the controlled experimental group showed a diffuse distribution. The fluorescence signal of fusion expression vector (35S:*GhBPC4*-GFP) was specifically enriched in the nucleus, but no observable signal was detected in the cytoplasm (Figure 7). The results confirmed that GhBPC4 is specifically localized to the nucleus, consistent with the subcellular characteristics of the transcription factor. This provides a structural basis for its molecular function in regulating the transcription of downstream target genes.

### 2.8. GhBPC4 Silenced Cotton Plants Showed High Sensitivity to Cold Stress

**Figure 8:**
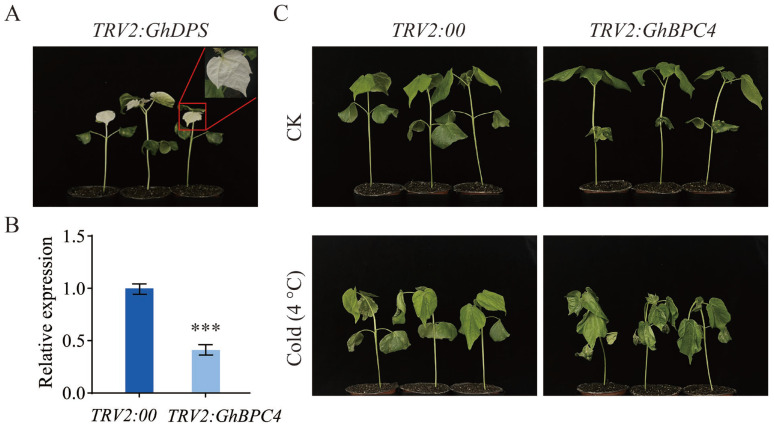
Effect of VIGS-mediated *GhBPC4* silencing on cotton cold tolerance. (**A**) Positive control albino phenotype of *TRV2:GhPDS* plants. (**B**) *GhBPC4* gene expression levels in silenced plants. (**C**) *TRV2:GhBPC4* plant phenotype analysis. Morphological changes in *GhBPC4*-silenced plants and control plants 48 h after cold stress (4 °C). Error bar represents ± SD (*n* = 3). Significance analysis by Student’s *t* test, with statistical significance denoted by *** *p* < 0.001.

To elucidate the role of *GhBPC4* in cotton cold stress response, the VIGS technique was used to construct a *GhBPC4*-silenced plant, and a no-carrier control (*TRV2:00*) were used as controls for functional verification. After 10 days of infection, *GhPDS*-silencing strains exhibited a typical bleaching phenotype (Figure 8A), indicating that the TRV vector-mediated gene silencing system operated efficiently. RT-qPCR assays showed that the expression level of the target gene *GhBPC4* in *TRV2:GhBPC4* plants was significantly downregulated compared with that in the *TRV2:00* control group (*p* < 0.001), and silence efficiency reached 60% (Figure 8B). Under 4 °C low-temperature stress (Figure 8C), silenced plants showed obvious damage symptoms such as severe dehydration and wilting, dry curling of leaves, tissue browning, and macula or necrotic spots on the surface after 48 h cold treatment. These findings demonstrate that suppressing *GhBPC4* expression via gene silencing markedly compromised the physiological adaptation of cotton plants to low-temperature stress, thereby underscoring its pivotal role in cold tolerance regulation.

## 3. Discussion

The BPC transcription factor family, as a plant-specific regulatory factor, is widely conserved across diverse angiosperms and found in *Arabidopsis thaliana*, *Solanum tuberosum*, *Hordeum vulgare*, *Cucumis sativus*, *Solanum lycopersicum*, *Gladiolus*, *Glycine max*, *Camellia japonica*, *Malus pumila*, and other plants [12,22,23,24,25,26,27]. Accumulating evidence demonstrates that the BPC transcription factor family orchestrates developmental processes and serves as a central regulator in biotic/abiotic stress responses by modulating downstream target genes [7,11]. Although the function of the BPC transcription factor family in plant stress response has been preliminarily reported, the systematic identification of this gene family in cotton (including member classification and conserved domain evolution) and its molecular regulatory network in response to low-temperature stress have not been systematically resolved yet. Employing *AtBPC* genes as queries, this study systematically identified orthologous members of the BPC transcription factor family across 10 *Gossypium* species. The analysis delineated conserved structural domains, phylogenetic clades, and gene duplication events, while transcriptomic profiling further elucidated tissue-specific expression and abiotic-stress-responsive regulatory patterns of *GhBPC* genes in *G. hirsutum*.

Four members of the *BPC* family were identified in diploid cotton, and eight were identified in tetraploid cotton (Table S1). The BPC transcription factor family typically retains 4–6 members in monocots (*Oryza sativa*), a distribution highly consistent with the 6–8 members observed in eudicots (*Dioscorea alata* L.). This numerical conservation across plant lineages stems from restricted gene duplication events, reflecting functional stability during the evolution of the *BPC* family in the plant kingdom [8]. This study found that all *BPC* members are unstable, which seems to conflict with the traditional ‘structure–function’ paradigm but actually reveals a new regulatory logic for plant stress responses. Traditional structural biology holds that a protein’s function depends strictly on its stable three-dimensional folding configuration. However, recent studies have confirmed that the widespread existence of Intrinsically Disordered Proteins (IDPs) or Intrinsically Disordered Regions (IDRs) challenges this paradigm, and this disorder is the core mechanism for achieving multifunctional regulation [28,29]. The characteristics of *BPCs* discovered in this study are highly consistent with the theory of IDPs. In eukaryotes, IDPs are ubiquitous. About 30% of eukaryotic proteins are completely disordered [30]. These disordered regions play key roles in transcriptional regulation, signal transduction, and cell cycle control. Their disorder confers conformational plasticity, allowing single proteins to bind multiple targets through different folding states [31]. The “unstable” properties of *BPCs* are not functional defects, but reflect the evolutionary advantages of IDPs. The synergistic effect of multiple disordered motifs achieves functional optimization through conformational plasticity. This is an adaptive evolution strategy that may enhance their adaptability to abiotic stress. Phylogenetic analysis classified cotton *BPCs* into three evolutionary subgroups (Figure 2), consistent with the taxonomic pattern of *A. thaliana BPCs* [9]. Members of the same subgroup exhibit highly similar exon–intron architectures and conserved protein motifs (motif 1), suggesting functional conservation within the subgroup (Figure 3), while significant differences in gene structure between subpopulations suggest that functional differentiation may be due to specific variations in evolutionary processes [32]. All BPC proteins carry a conserved GAGA-binding domain that specifically recognizes GA-rich *cis*-acting elements, which is the structural basis for their transcriptional regulatory functions [32,33]. *AtBPC6* regulates epigenetic modification by recruiting the PRC1/PRC2 complex by binding to the GAGA domain [9]. Collinearity analysis found that segmental replication is the main form of gene expansion in the WGD event of cotton (Figure 5). This replication pattern may enhance the adaptability of plants to environmental stress through functional redundancy while enhancing genomic plasticity. This is consistent with the evolution strategy of plants to cope with stress [34].

Transcriptional regulation dynamically coordinates plant growth and developmental processes through interplay between *cis*-acting elements and transcription factors, while triggering stress-specific responses under abiotic challenges such as drought, low-temperature, or high-salinity conditions [35]. The combinatorial architecture and quantitative profile of *cis*-acting elements constitute fundamental determinants of transcriptional initiation complex assembly efficiency and stability, thereby defining hierarchical gene expression regulatory networks [36]. The promoter regions of *BPC* genes harbor combinatorial cis-regulatory modules (JA-responsive CGTCA-motif, ABA-dependent ABRE, antioxidant defense-associated ARE, drought-inducible MBS, and light-signaling G-box), which integrate phytohormone signaling (MeJA/ABA) with stress response pathways to dynamically orchestrate morphogenesis, abiotic stress tolerance, and photoadaptation in cotton. (Figure 4). Consistent with the tomato study, an orthologous MBS *cis*-regulatory element (MYB-binding site) was identified in the promoter regions of PLATZ family transcription factors. This element recruits MYB transcription factors to activate downstream stress-responsive genes, thereby playing a pivotal role in abiotic stress adaptation [37]. Combined with the results of differences in the expression levels of the *GhBPC* gene in different tissues and abiotic stresses (Figure 6), it can be speculated that *BPC* genes may have important functions in plant growth and development and environmental stress responses. This is consistent with the study of the *B. napus BBR-BPC* family. There are tissue-specific differences in expression patterns, and different members also have different expression patterns under different abiotic stresses, playing an active role in coping with salt and drought stress. RT-qPCR assays revealed significant transcriptional levels of *GhBPC4* under cold stress, implicating its pivotal role in cotton cold adaptation. Similarly, tomato *SlBPC2* exhibited transient upregulation at 3 h post-cold-treatment, thereby reflecting conserved functions of *BPC* members in early-stage cold signal transduction. [19]. Enrichment of hormone-responsive cis-elements in the *GhBPC* promoter region mediates the recruitment of transcription factors that dynamically integrate the ABA-JA-GA signaling pathway to coordinate cotton reproductive-stage switching. This mechanism is consistent with the known function of the *A. thaliana* homologous gene *AtBPC6* [18], and in *B. napus*, members of the *BnBBR-BPC* family exhibit differential expression patterns in hormone stress responses, with *BnBBR-BPC25* playing a key regulatory role. Normally, most transcription factors are localized in the nucleus. This study confirmed that GhBPC4 is localized in the nucleus (Figure 7), consistent with other *BPC* homologues, and similar localization results were observed in *SlBPC* and *CjBPC1* genes [19]. These findings suggest that *BPC*-homologous genes of different species may have conserved functions in abiotic stress responses. The results of this study further suggest that *GhBPC4*-silenced plants exhibit more severe leaf wilting, drying, and curling symptoms than control plants under cold treatment conditions (Figure 8). This phenotypic feature is consistent with that of cold-sensitive plants reported in *NtBBX9*- and *SgNAC1*/*SgNAC2*-gene-related studies, both of which showed different degrees of leaf wilting [38,39].

This study provides a comprehensive evolutionary and functional characterization of the cotton *BPC* gene family, identifying *GhBPC4* as a pivotal regulator of cold stress adaptation. In future work, transgenic lines can be constructed by transgenic technology to further explore the functional redundancy and synergistic mechanism of members of different subfamilies. At the same time, it provides a theoretical basis for crop stress resistance breeding.

## 4. Materials and Methods

### 4.1. Plant Materials and Experimental Treatments

The germplasm of plant material used in the experiment was *G. hirsutum*; the cotton seeds were delinted with sulfuric acid and sown in a nutrient bowl filled with nutrient soil. Plants were maintained in controlled environment chambers with thermostatic regulation at 25 °C (±2 °C tolerance range) and diurnal rhythm programming of 16L:8D (light/dark ratio). Fifteen-day-old vegetative-stage plantlets were exposed to 4 °C treatment in growth chambers, maintaining standardized photoperiodic parameters. Leaf samples were collected at different time points (0 h, 1 h, 3 h, 6 h, and 12 h following stress induction) after treatment, then freeze-fixed immediately to maintain RNA integrity at −80 °C until nucleic acid extraction.

### 4.2. Identification of Cotton BPC Family Members

Comprehensive genomic datasets for *Gossypium* species were retrieved from CottonMD, and a local BLAST (v2.14.0) database was created including the following *Gossypium* species: A chromosome group: *G. herbaceum* (WHU, A1), *G. arboretum* (CRI, A2); D chromosome group: *G. thurberi* (ISU, D1), *G. raimondii* (NSF, D5), *G. turneri* (NSF, D10); AD chromosome group: *G. hirsutum* (JGI, AD1), *G. barbadense* (H7124,AD2), *G. tomentosum* (HGS, AD3), *G. mustelinum* (JGI, AD4), *G. darwinii* (HGS, AD5) [40]. Seven *Arabidopsis thaliana AtBPC* sequences were retrieved from the TAIR database as queries, and homologous sequences were systematically screened across the genomic datasets of ten cotton species using BLASTP (v2.14.0) [41]. An E-value threshold of 1.0 × 10^−20^ was established to minimize false positives, with all other parameters set as defaults. Initial candidate sequences from the search were profiled against the NCBI-CDD (v3.21) to identify *BPC*-specific conserved domains. Only sequences harboring intact domains were retained as members of the family, while sequences with ≥90% amino acid identity were considered redundant. We retained the longest sequences as representative members and combined gene structural conservation and domain integrity to eliminate sequences with fragments or structural abnormalities [42]. Chromosomal coordinates of members were parsed from genome annotation files (GFF3 format) using TBtools (v2.210), with chromosomal-scale metrics obtained via the Fasta Stats module. Spatial distributions of these genes across ten *Gossypium* species were subsequently visualized on chromosomes using the MG2C platform [43]. *BPC* family genes were named based on chromosomal positions. Naming conventions were based on the logic of species abbreviations, family member numbers, and chromosome locations. Physicochemical properties of their protein sequences were characterized using the Protein Parameter Calc module in TBtools software.

### 4.3. Phylogenetic Analysis of BPC Gene Family

Protein sequences of the *BPC* gene family from *Gossypium* species, *A. thaliana,* and *Theobroma cacao* were aligned using the ClustalW module within MEGA-X (v10.2.6) (default settings). Phylogenetic relationships were reconstructed via the Neighbor-Joining (NJ) algorithm with 1000 bootstrap replicates for node reliability assessment [44]. The resultant phylogenetic tree was optimized using the iTOL (v6.0) online interactive platform [45].

### 4.4. Comprehensive Characterization of Genomic Architecture, Conserved Motifs, and Protein Domains in BPC Gene Family

To analyze the gene structure, conserved domains, and conserved motifs of the *BPC* gene family, the following procedures were implemented: The conserved motifs of BPC protein sequences were first identified using the MEME Suite online platform, with the maximum number of motifs set to 10 and other parameters maintained as defaults [46]. Subsequently, integrated visualization of conserved motifs and gene structures was performed via the “Gene Structure View (Advanced)” package in TBtools software, followed by refinement of graphical elements using Adobe Illustrator (v27.0.0.602).

### 4.5. Systematic Identification and Regulatory Role of Cis-Acting Elements Within BPC Gene Family

Promoter regions spanning 2000 bp upstream of the ATG start codon for each *BPC* gene family member were extracted using TBtools. Subsequently, all obtained promoter sequences were analyzed via the PlantCARE web server to identify *cis*-regulatory elements and characterize their distribution patterns [47]. The predicted *cis*-regulatory elements were then visualized using TBtools.

### 4.6. Interspecies Collinearity Analysis and Visualization

Interspecies collinearity among ten cotton genomes was analyzed using the One Step MCScanX (v1.3.0) module in TBtools, followed by visualization of collinear relationships through the Advanced Circos package (v2.085) [48].

### 4.7. GhBPC Expression Pattern Analysis

Transcriptomic datasets encompassing multiple tissues and abiotic stress conditions of *G hirsutum* were analyzed to profile *BPC* gene expression patterns [49]. The transcriptomic dataset analyzed in this study was obtained from a public database with a login number of PRJNA248163 obtained from the NCBI SRA database. Heatmaps were generated using the Heatmap module in TBtools, with transcriptomic data standardized by Log_2_(FPKM+1). For experimental validation, RT-qPCR assays were performed using *GhUBQ7* as the internal reference gene, and relative expression levels were calculated via the 2^−ΔΔCt^ method [50]. Primer sequences are provided in Supplementary Table S2.

### 4.8. Subcellular Localization Analysis of GhBPC4 Protein

*GhBPC4* was amplified via PCR and cloned into the pCAMBIA1300-GFP expression vector through homologous recombination, yielding a 35S:*GhBPC4*-GFP fusion construct (primers listed in Supplementary Table S2). The empty pCAMBIA1300-GFP vector and a nuclear-localized marker vector (pCAMBIA1300-35S-mCherry-NLS) served as negative and positive controls, respectively. Recombinant plasmids were introduced into *Agrobacterium tumefaciens* GV3101 competent cells via freeze–thaw transformation. Bacterial pellets were centrifuged and resuspended in infiltration buffer to an OD_600_ of 0.6. Bacterial suspensions were injected into the abaxial epidermis of young leaves from 4–5-week-old *Nicotiana benthamiana* plants using a needleless syringe. Infiltrated plants were dark-incubated for 24 h, followed by cultivation under standard conditions (25 °C) for 48 h. Subcellular localization of GhBPC4 was determined by observing GFP fluorescence in infiltrated leaf regions with a confocal laser scanning microscope (excitation wavelength: 488 nm).

### 4.9. Functional Validation via Virus-Induced Gene Silencing in Cotton

To characterize candidate gene functions, a Tobacco Rattle Virus (TRV)-based Virus-Induced Gene Silencing (VIGS) system was established in upland cotton (*G. hirsutum*). The phytoene synthase gene (*GhPDS*) served as a positive control, whose silencing generated visible photobleaching phenotypes for rapid system validation [51,52]. Target-specific fragments of *GhBPC4* were PCR-amplified and cloned into the TRV2 vector via homologous recombination, with empty vector *TRV2:00* as a negative control (primers in Supplementary Table S2). Recombinant plasmids and helper vector TRV1 were introduced into GV3101 competent cells using freeze–thaw transformation. Bacterial pellets were centrifuged and resuspended in infiltration buffer to OD_600_ = 1.0. TRV1 was mixed with each TRV2 recombinant at a 1:1 volumetric ratio, followed by 3 h of dark incubation. Cotyledons of 8-day-old seedlings with fully expanded leaves were agroinfiltrated, then dark-incubated at 22–25 °C for 24 h before transfer to standard growth conditions (25 °C). Successful VIGS operation was confirmed by photobleaching in *TRV2:GhPDS* plants. Total RNA from leaves of *TRV2:00* and *TRV2:GhBPC4* plants was subjected to RT-qPCR to evaluate *GhBPC4* knockdown efficiency. Functional validation of *GhBPC4* in cotton cold stress adaptation was performed by subjecting both gene-silenced plants and wild-type controls to 4 °C low-temperature treatment.

### 4.10. Data Statistical Analysis

Data analysis and visualization were performed using GraphPad Prism (v10.1.1). All experiments in this study were set up with 3 biological replicates, and the difference analysis was performed by Student’s *t* test and one-way ANOVA (95% confidence interval), respectively. The significance levels are indicated by asterisks, and the error bars in all graphs indicate the mean square error (SD). The statistical significances were * *p* < 0.05, ** *p* < 0.01, and *** *p* < 0.001, respectively.

## 5. Conclusions

This study performed genome-wide identification of *BPC* gene family members across 10 *Gossypium* species, followed by multidimensional characterization of their physicochemical properties, evolutionary relationships, chromosomal distributions, *cis*-regulatory elements, conserved domains, collinearity networks, and expression profiles. *GhBPC4* was established as a key regulator governing cold stress responses in cotton. Critically, VIGS assays demonstrated that *GhBPC4* knockdown substantially compromises cold tolerance. These breakthroughs deepen mechanistic insights into *BPC* gene functions in cotton, simultaneously providing pivotal targets for molecular breeding and foundational biological research.

## Data Availability

Data is contained within this article or the Supplementary Materials.

## Supplementary Materials

The following supporting information can be downloaded at: https://www.mdpi.com/article/10.3390/ijms26167978/s1.
